# Transmission and Persistence of Infant Gut-Associated Bifidobacteria

**DOI:** 10.3390/microorganisms12050879

**Published:** 2024-04-27

**Authors:** Margaret A. Hilliard, David A. Sela

**Affiliations:** 1 Department of Food Science, University of Massachusetts, Amherst, MA 01003, USA; hilliard@umass.edu; 2 Organismic and Evolutionary Biology Graduate Program, University of Massachusetts, Amherst, MA 01003, USA; 3 Department of Nutrition, University of Massachusetts, Amherst, MA 01003, USA; 4 Department of Microbiology, University of Massachusetts, Amherst, MA 01003, USA; 5 Department of Microbiology & Physiological Systems and Center for Microbiome Research, University of Massachusetts Medical School, Worcester, MA 01605, USA

**Keywords:** *Bifidobacterium infantis*, bifidobacteria, human milk, infant gut microbiome

## Abstract

*Bifidobacterium infantis* are the primary colonizers of the infant gut, yet scientific research addressing the transmission of the genus *Bifidobacterium* to infants remains incomplete. This review examines microbial reservoirs of infant-type *Bifidobacterium* that potentially contribute to infant gut colonization. Accordingly, strain inheritance from mother to infant via the fecal-oral route is likely contingent on the bifidobacterial strain and phenotype, whereas transmission via the vaginal microbiota may be restricted to *Bifidobacterium breve*. Additional reservoirs include breastmilk, horizontal transfer from the environment, and potentially in utero transfer. Given that diet is a strong predictor of *Bifidobacterium* colonization in early life and the absence of *Bifidobacterium* is observed regardless of breastfeeding, it is likely that additional factors are responsible for bifidobacterial colonization early in life.

## 1. Introduction

**Figure 1:**
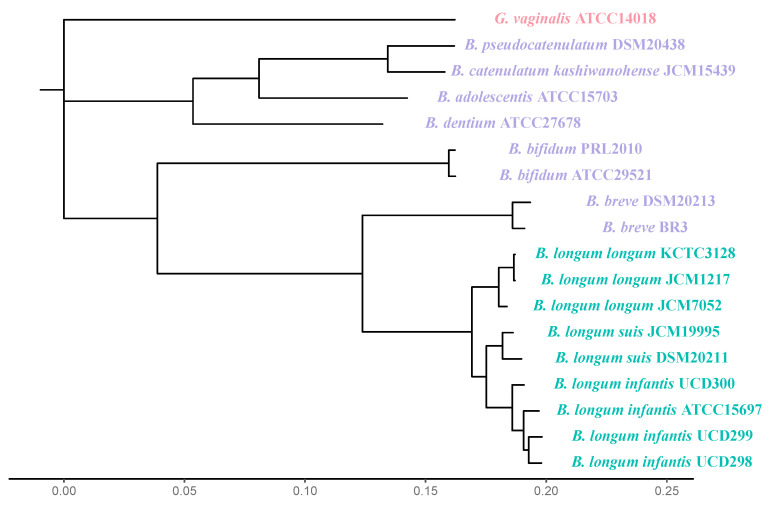
An 81 single-copy core gene phylogeny of human gut-associated *Bifidobacterium* strains (Supplementary Table S1) shows the evolutionary relationships within the *B. longum* subspecies (labeled in green), relative to other infant-associated *Bifidobacterium* species commonly isolated from infant feces with varied abilities to utilize human milk oligosaccharides (labeled in purple). The outgroup is labeled in red.

**Figure 2:**
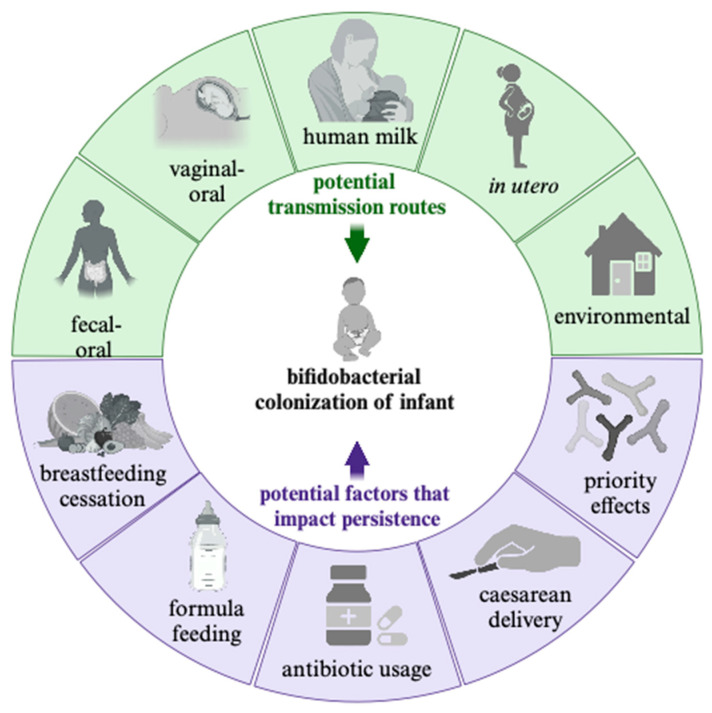
Potential transmission routes and persistence factors contributing to bifidobacterial colonization of the infant gut. Transmission routes are depicted in green, whereas the factors that impact bifidobacterial persistence during early life are indicated in purple. Created with BioRender Scientific Illustration Software.

The human gut microbiota contains trillions of microbial cells, most densely populating the lower gastrointestinal tract (GIT) [1]. The composition and function of the various microbial communities along the GIT stabilize to resemble that of an adult-like community within the first three years of life, a process guided by birth and feeding modality, antibiotic usage, and environmental exposures, among other factors [2]. *Bifidobacterium longum* is a high-G+C Gram-positive anaerobic bacterium that colonizes the distal GIT of mammalian hosts as well as other animals, where they utilize host-indigestible carbohydrates as a fermentative carbohydrate source [3,4]. In human infants, *Bifidobacterium* species that utilize human milk oligosaccharides (HMOs) are often dominant, where they could represent 50–70% of the bacterial taxa within the infant gut microbiome [5,6]. HMOs are the third most abundant component in human milk without a direct nutritive value to infants, who are unable to digest them [7,8]. Several *Bifidobacterium* species have been identified and isolated from infants with varied abilities to utilize HMOs, typically comprised of *Bifidobacterium longum* subsp. *infantis*, *Bifidobacterium longum* subsp. *longum*, *Bifidobacterium bifidum*, *Bifidobacterium breve*, *Bifidobacterium catenulatum* subsp. *kashiwanohense*, and *Bifidobacterium psudocatenulatum* (Figure 1) [9]. A growing body of evidence suggests that the representation of certain infant-type bifidobacteria are diminished in Western infant populations, potentially due to a reduction in breastfeeding rates [10]. This reduction in bifidobacterial representation, however, is also observed in breastfed infants, indicating that there are likely additional factors at play (Figure 2) [11,12]. This review summarizes and integrates the evidence regarding microbial reservoirs of infant-type *Bifidobacterium* outside of the infant gut, their associated transmission routes, and population persistence with a particular focus on *B. longum* subsp. *infantis* (herein *B. infantis*).

## 2. Physiological Significance of *B. infantis* in the Infant Gut Microbiota

*B. infantis* exhibit unique genotypic and phenotypic properties that provide a fitness advantage within the ecology of the infant GIT and thus are likely adaptive consequences of co-evolution with the nursing infant. Accordingly, the *B. infantis* chromosome features co-linear locus encoding proteins dedicated to the binding, import, and intracellular degradation of HMOs as a carbohydrate source [13]. In addition, *B. bifidum* and *B. breve* exhibit remarkable conservation of genomic features that contribute to the HMO utilization phenotype and deploy intracellular and extracellular glycolytic strategies dependent on the taxon or strain [14]. Dependent on the strain, *B. breve* utilizes constituent residues of HMO released by other *Bifidobacterium* species, such as *B. bifidum* in co-cultivated in vitro conditions [15] and could utilize HMOs with homologous mechanisms deployed by *B. infantis* [16]. 

HMOs added to formula milk increase *B. infantis* abundance in the infant gut, which more closely mirrors the human milk-fed infant gut microbiome [17]. A major line of scientific inquiry is dedicated to characterizing the physiological role of *B. infantis* dominance within the infant gut, as some host benefits are species- and strain-specific [18,19]. Much of this is centered on soluble molecules secreted by *B. infantis* while metabolizing HMOs, including the end products lactate and acetate [20]. These organic acids lower the infant’s gut pH and protect against pathogen colonization [21]. The ratio of lactate to acetate is an indicator of metabolic efficiency for bifidobacterial cellular operations, and once secreted, they are responsible for initiating a downstream cascade of syntrophic interactions [20,22]. In addition, *B. infantis* colonization normalizes intestinal barrier function in a mouse model of colitis and potentially in human infants [23]. Significantly, intestinal barrier dysfunction is associated with inflammatory and metabolic disorders [24,25]. 

Compositional differences and altered diversity of the intestinal microbiota in early life, largely attributed to feeding mode, are associated with pathologies in vulnerable infant populations, including necrotizing enterocolitis, allergies, atopy, obesity, and type 1 diabetes [26,27,28,29]. Currently, there are not clear in vivo causal mechanisms for infant-type *Bifidobacterium* mitigating the development of these disease states. There are, however, in vitro and ex vivo studies that demonstrate the benefits of *Bifidobacterium* species, including *B. infantis*, such as converting amino acids to indole-lactate which reduces the inflammatory response in immature cell lines and is linked with *B. infantis* ability to utilize HMOs [30,31]. Taken together, *B. infantis* is a specialized mutualist within the infant gut microbiome that supports homeostatic processes within the developing neonate. Much of this is due to the intracellular transport and utilization of HMOs and the subsequent secretion of metabolic end products available to the heterogenous gut microbial community and host [32]. These functions are intimately linked with the proliferation of bifidobacterial populations during breastfeeding and are largely attributed to the ability to utilize HMOs secreted during nursing. 

## 3. Patterns of *Bifidobacterium* Distribution across Infant Populations

The diversity of *Bifidobacterium* species representation in the infant gut is currently understood to be dependent on geography, nutrition, and environmental factors. Although the relative contributions of these parameters are not scientifically defined with predictive power. One current model, albeit limited, postulates that infants that live in industrialized societies are colonized with a lower relative abundance of *B. infantis* and are colonized instead by *B. breve*, *Bifidobacterium adolescentis*, or *B. longum* subsp. *longum* (Figure 1) [10,33]. In addition, the model postulates that microbial succession in industrialized societies may follow a stochastic model marked by a relative increase in diversity over time associated with the cessation of breastfeeding [33,34]. Interestingly, *B. infantis* may be negatively correlated with *B. breve*, suggesting that they compete for a similar niche within the infant gut [34]. The latter facilitates a shift to an adult-like gut microbial composition after two years and is negatively associated with *B. longum* in adulthood in a cohort of Norwegian infants [35]. 

*B. infantis* achieves relative abundances potentially greater than 50% in geographic regions and cultural groups with relatively high rates and durations of breastfeeding [10]. There is variation in *Bifidobacterium* abundance within and between infant populations, with cohorts that occupy the same geographical location exhibiting more similar compositions to each other than expected by stochastic distribution [36]. As an example, 75% of *Bifidobacterium* that colonized healthy breastfed Bangladeshi infants in the first 12 months of life were identified as *B. infantis*, whereas the relative proportions of other bifidobacteria increased over time [37]. In contrast, infants are colonized by several bifidobacterial taxa in regions with low historical rates and durations of breastfeeding, rather than one dominant species [10], reflecting a more adult-like microbiota profile [37]. Interestingly, infant populations could feature low representation or a complete absence of *B. infantis* in the gut microbiome during infancy [11,38,39]. Accordingly, *B. infantis* was differentially prevalent in two populations living in relative proximity to one another in New York, USA [38], where *B. infantis* in the rural population was similar to those in developing countries. This suggests that lifestyle and cultural factors contribute to the relative abundance and prevalence of *B. infantis*, not geography alone. 

The prevailing hypothesis is that vertical transmission of microbiota during birth explains bifidobacterial acquisition early in life. Horizontal routes of colonization may be secondary in importance or replace vertical transmission as the primary path in certain contexts or populations. Regardless, the mechanisms underpinning the origin and transmission of *B. infantis* remain incompletely characterized. In addition, recent scientific scrutiny challenges the understanding that the womb is sterile and thus provides a potential route of colonization [40]. 

## 4. Microbial Reservoirs of Infant-Type *Bifidobacterium* and Related Hypotheses for *Bifidobacterium* Transfer to the Infant

The physiological ability of *B. infantis* to consume HMOs efficiently requires initial, or serial, inoculation of relevant populations. Although a rigorous identification and characterization of *B. infantis* environmental reservoirs remain elusive, infant-type *Bifidobacterium* are isolated from ecological niches other than infant stool.

### 4.1. Maternal Fecal-Oral Transmission of Bifidobacterium

The fecal-oral route is the most supported mechanism of vertical transmission of maternal *Bifidobacterium* to infants in early life due to the viability and persistence of strains in stool [41]. In adulthood, *Bifidobacterium* is a relatively low abundance taxa that often colonize below detection limits [42], thus posing a challenge to describe the reservoirs of *Bifidobacterium* completely and accurately within adult populations. Species such as *B. longum* and *B. adolescentis* were the most abundant species in a cohort of Irish mothers, at approximately 2% relative abundance within stool [43]. *B. infantis*, in contrast, were quantified in maternal stool in a Japanese cohort but were not the most abundant bifidobacterial species [44]. Although *Bifidobacterium* colonizes the adult gut to a lesser extent than infants, progesterone may regulate the abundance of *Bifidobacterium* during pregnancy, which could facilitate transmission to the infant during birth [43,45]. 

Several small cohort studies demonstrate that *B. longum* and *B. infantis* strains are transmitted from mother to infant via stool in a small percentage of participants [46,47,48,49]. This is somewhat confounded by the phylogenetic similarity between *B. longum* and *B. infantis*, which could make discriminating between them challenging even with molecular methods (Figure 1). Similarly, *B. bifidum* and *B. breve* strains were shared between mother and infant stools at 10 days of life, but only *B. bifidum* was detected in infant stool samples by three months [35]. Notably, viable but not culturable bacteria can escape detection due to differences in media and selectivity, which reduces the efficacy of culture-dependent studies. A non-dominant maternal strain of *Bacteroides uniformis* was inherited when the strain contained functionally advantageous starch utilization loci [50], which may aid in sialylated milk oligosaccharide utilization in the infant gut [51]. Taken together, strain inheritance from mother to infant via the fecal-oral route is contingent on the phenotypic capabilities of the strain being transmitted to the infant. 

### 4.2. Maternal Vaginal-Oral Transmission of Bifidobacterium

*B. infantis*, *B. longum*, and *B. bifidum* are seldom detected in the vaginal canal, and strains are rarely congruent between the fecal and vaginal microbiota during pregnancy [44,52]. Thus, the vaginal canal has been investigated as a potential source of *Bifidobacterium* that colonizes infants in early life. This is consistent with the conventional understanding that infants born vaginally acquire microbiota resembling the vaginal canal, whereas infants born via Cesarean section harbor microbes resembling skin microbiota [53]. Several studies have concluded that vertical transmission via fecal transfer during vaginal births is a more critical source of *Bifidobacterium* but found that *B. breve* from the vaginal environment colonized the infant gut microbiota [43,44]. Interestingly, longer maternal labor was negatively correlated with bifidobacterial abundance [54], suggesting that labor duration may be a factor that influences the transmission of *Bifidobacterium* during vaginal births.

### 4.3. Maternal Transmission of Bifidobacterium *via* Human Milk

The human milk microbiome is compositionally similar to the infant oral microbiota, yet maternal milk correlates to the microbial composition of infant stool [55]. Viable *Bifidobacterium* is often difficult to isolate from human milk due to its low abundance, which is considered close to the detection limit for culture-based methods [56]. In most instances, however, bifidobacterial DNA signatures are more readily detectable by molecular methods [57]. Nevertheless, viable *B. longum*, *B. breve*, *B. adolescentis*, *B. bifidum*, and *B. pseudocatenulatum* have been isolated from human milk, albeit often in a small percentage of study participants [47,57,58]. Furthermore, *B. longum* strain congruence between mother’s stool before delivery with those in breastmilk and infant stool were identified in a small portion of a Belgian cohort [48]. Similarly, *B. longum* and *B. breve* strain congruence was demonstrated in a cohort from Spain between human milk and corresponding infant stool samples [59]. 

Several factors could potentially contribute to the abundance of bifidobacteria present in human milk. For example, *Bifidobacterium* is more frequently detected in milk from mothers who delivered vaginally [60]. In addition, *Actinobacteria* were positively associated with directly nursing the infant, suggesting that pumped or stored milk may not be a comparable source of *Bifidobacterium* [61]. *Bifidobacterium* are detected in greater abundance in human milk late in lactation and during exclusive breastfeeding, and therefore, it is speculated that these microbes originate from retrograde flow from the infant’s oral cavity [60,62,63]. As a result, it remains unclear if human milk facilitates the acquisition of *Bifidobacterium* or if it plays a transient and supportive role in *Bifidobacterium* transmission to infants in early life. 

### 4.4. Maternal Transmission of Bifidobacterium in Utero

The dogmatic view that the uterine environment is invariably sterile has been scientifically revisited [64], although several methodological challenges to fully characterize the system remain [65]. Nevertheless, most evidence for in utero colonization of *Bifidobacterium* is obtained from animal models. In one study, rat dams were fed either a single- or multi-strain probiotic and delivered their pups via Cesarean section. Compellingly, genomic DNA residues from probiotic strains were detected in maternal placental tissue, although the strains themselves were not culturable [66]. Another study administered a strain of *Enterococcus faecium* to pregnant mouse dams that were labeled with a uniquely identifiable insertion sequence. The labeled strain was subsequently identified in pup meconium after Cesarean-section delivery [67]. In a human study, identical strains of *B. breve* were identified in maternal vaginal and infant stool samples following C-section delivery, which implicates breastmilk or prenatal transmission [40]. DNA sequencing is much more sensitive than culture-based techniques, although low-abundance taxa and their nucleic acid signatures may evade characterization and are prone to confounding contamination from the human subject, lab reagents, and study personnel. Accordingly, negative controls have been demonstrated to harbor similar microbial DNA as placental samples [68], reflecting the challenge of incisively defining the “placental microbiota”. 

### 4.5. Post-Partum Horizontal Transmission of Bifidobacterium Remains an Avenue for Future Research

Strain sharing is evident in families, irrespective of delivery type, which suggests that post-partum horizontal transmission of host-associated microbes occurs and may play a role in early life colonization [69]. In cases where infants are not vaginally delivered or breastfed, horizontal transfer from family members, including older siblings, remains the most parsimonious explanation for infant-type *Bifidobacterium* colonization outside of probiotic administration. Thus, infants may rely on contact with other breastfed infants to acquire *B. infantis* [10], but this hypothesis has not been empirically tested and remains an avenue for future research. 

*Bifidobacterium* suspended in house dust due to aerosolization of fecal material during routine caregiving may be a source of inoculation early in infant gut colonization and potentially a reservoir for reintroduction after microbiome disruption due to infection or antibiotic usage [70,71]. Similarly, *Bifidobacterium dentium* was found in higher abundance in salivary samples from pregnant women than in menstruating women [72], which potentially serves as an inoculation source for infants during contact and caregiving. Any contributions made by *B. dentium* to infant gut microbiome function would likely be as an allochthonous member through transient passage. Regardless, oral transmission is a route for *Bifidobacterium* colonization of the infant gut microbiota [73], but to what degree horizontal transfer plays in *Bifidobacterium* acquisition in infancy warrants further investigation. 

## 5. Additional Factors That May Influence *Bifidobacterium* Persistence Early in Life

### 5.1. Breastfeeding and Dietary Glycans Maintain Bifidobacterium Populations

The HMO fraction of human milk selects infant-type *Bifidobacterium* with the ability to utilize HMOs, and thus breastfeeding maintains the bifidobacterial population in early life [74,75]. Similarly, the cessation of breastfeeding is implicated in the succession of microbial taxa and the subsequent reduction in the relative abundance of infant-associated *Bifidobacterium* rather than the introduction of solid and complementary foods [76]. In line with this claim, a transitionary clade of *B. infantis* thrived during periods of mixed feeding in populations with high rates of breast feeding [77]. In addition, a diet low in host-indigestible carbohydrates over several generations leads to the irreversible loss of gut microbial diversity in a rodent model [78]. These findings suggest that current and historical feeding practices drive the maintenance of *Bifidobacterium* in individuals and populations. In addition, it is tempting to speculate that infant diets deficient in glycans have driven a dramatic reduction in endogenous *Bifidobacterium* in populations with low historical rates and durations of breastfeeding [79]. 

### 5.2. Diet Is a Longitudinal Predictor of Bifidobacterium Colonization in Early Life

Administration of intrapartum antibiotics reduces bacterial transmission in vaginally delivered infants [69] and is associated with reduced *Bifidobacterium* populations in the infant gut among vaginally delivered and breastfed infants at three months [80]. Breastfeeding for more than six weeks partially mitigates compositional differences seen at 6 weeks of life associated with birth mode at one year [81]. Therefore, infant feeding may be more predictive of gut microbial composition regardless of birth mode after one year, but it remains unclear if *Bifidobacterium* species are specifically implicated. The importance of early transmission and development of the infant gut microbiota in the first year of life is not discounted, as a decrease in stool microbial diversity in the first three months of life is correlated with the development of allergies during early life or school age [82,83]. Interestingly, there are associations between early childhood allergic disease development and alterations in the skin and lung microbiomes during infancy [84,85]. 

Infant-derived *Bifidobacterium* have been shown to colonize rat and mouse guts more efficiently when multiple strains are present [66,86]. Thus, one speculates that the historical loss of these microbes driven by modern delivery practices and hygiene may have reduced early life transmission, and this loss may limit the polymicrobial cooperative interactions of *Bifidobacterium* and result in a subsequent reduction in competitive advantage. 

### 5.3. How Do Some Bifidobacterium Species Dominate the Microbiome at the Expense of Others?

Despite *Bifidobacterium* being regarded as the primary colonizer of the infant gut, only recently have bifidobacterial succession patterns and their carbohydrate phenotypes been linked to community structure [87]. As an example, *B. infantis* and *B. bifidum* have inhibitory priority effects on other members of the *Bifidobacterium* genus in vitro. Accordingly, *B. infantis* has a large complement of bacteriocin gene clusters relative to other *Bifidobacterium* species, which may contribute to its competitive advantage within the infant GIT [88]. In addition, bifidobacterial species and strains differentially secrete exopolysaccharides that help evade immune responses, promote stress tolerance and persistence in the gut, and possess antibacterial properties [89,90]. Interestingly, exopolysaccharides produced by *Enterococcus faecalis* (isolated from human milk) promoted the growth of *Bifidobacterium* species in vitro [91]. These results suggest that exopolysaccharide production and utilization may mediate *Bifidobacterium* colonization success in early life and that this function may be modulated through breastfeeding. 

### 5.4. What Taxa Potentially Fill the Open Niche When Bifidobacteria Are Absent? 

In the absence of *B. infantis* or other HMO-utilizing bifidobacteria, the open niche may be filled by mucin-degrading bacteria. *Lachnospiraceae* and *Bacteroidaceae* colonize infants in early life and degrade structural aspects of HMOs, partly due to structural similarities with mucins and the enzymatic machinery involved in their utilization [92,93,94]. Some *Bacteroides* species degrade or consume a variety of HMOs, although short-chain HMOs such as lacto-N-neotetraose (LNnT) select *B. infantis* over *Bacteroides thetaiotaomicron* in a mouse model. This suggests that if both species are present, the niche to degrade HMOs may be filled more efficiently by *B. infantis* [95]. In addition, *Lachnospiraceae* abundance tends to be greater in stool samples with low *Bifidobacteriaceae* abundance, regardless of diet [11]. Although, when *B. infantis* is administered as a probiotic to infants early in life, the relative abundance of *Lachnospiraceae and Bacteroidaceae* decreases [96]. 

## 6. Concluding Remarks

A diet rich in host-indigestible HMOs promotes *Bifidobacterium* colonization in early life, although the absence of *Bifidobacterium* is observed regardless of infant breastfeeding. As such, disruptions in microbial succession or initial inoculation of *Bifidobacterium* (e.g., antibiotic administration or delivery method via Cesarian section) influence colonization rather than diet alone (Figure 2). Consistent with previous hypotheses [10,79], we posit that reduced breastfeeding levels diminish HMOs in the infant diet to drive the reduction of *Bifidobacterium* in Western populations. Furthermore, a reduction in early life transmission events is imposed by modern hygienic practices, as well as delivery modes, and may further amplify the effect of diminished dietary HMOs. 

There is a pressing scientific imperative to characterize the transmission of bifidobacteria from potential reservoirs to infants with sufficient participants to enable conclusions with predictive value. In addition, there is a need to rigorously identify to what extent the mixed transfer of *Bifidobacterium* occurs at the strain level. It is the gold standard to determine congruence between bifidobacterial strains in their reservoirs and within the infant, although this necessitates the use of precise and often time-consuming methodology [97,98,99]. Nevertheless, the development of culture-based techniques with higher throughput in tandem with existing sequencing and computational approaches will strengthen future evidence in this regard. This will increase confidence in the hypothetical mechanisms by which the bifidobacterial population is acquired in early life. 

Additional empirical data are required to derive globally relevant scientific conclusions regarding bifidobacterial transfer. For example, the majority of microbiome studies in Africa are observational [100], which may not serve the needs of those communities. In addition, future studies will need to account for variation associated with race and ethnicity [74], as inequalities and health disparities may have a significant impact on early life feeding, gastrointestinal colonization, and lifelong health [101,102]. 

## Data Availability

The original contributions presented in the study are included in the Supplementary Materials, further inquiries can be directed to the corresponding author.

## Supplementary Materials

The following supporting information can be downloaded at: https://www.mdpi.com/article/10.3390/microorganisms12050879/s1, Table S1: Bifidobacterium strains and assession numbers used to construct the phylogeny. References [103,104,105,106,107,108,109,110,111,112] are cited in the Supplementary Materials.
