# Supplementary material for: Transmission and Persistence of Infant Gut-Associated Bifidobacteria
Source: Microorganisms. 2024 Apr 27;12(5):879. doi: 10.3390/microorganisms12050879 (PMC11124121; doi:10.3390/microorganisms12050879)
Supplement: Supplementary file 1 [file microorganisms-12-00879-s001.zip › Supplementary-Table S1.pdf]

Species designation	Strain Name	IMG Genome ID or GenBank accession	Isolation source	Additional information
Bifidobacterium adolescentis	ATCC15703	GCA_000010425.1	Human feces	
Bifidobacterium bifidum	ATCC29521	GCA_001025135.1	Human infant feces	JCM 1255; DSM 20456
Bifidobacterium bifidum	PRL2010	GCA_000165905.1	Human infant feces	
Bifidobacterium breve	BR3	GCA_001281425.1	Human infant feces	
Bifidobacterium breve	DSM20213	GCA_001025175.1	Human infant feces	JCM 1192; LMG 13208; ATCC 15700; NCTC 11815
Bifidobacterium dentium	ATCC27678	GCA_000172135.1	Human feces	
Bifidobacterium longum subsp. *infantis*	ATCC15697	643348516	Human infant feces	JCM 1222; DSM 20088
Bifidobacterium longum subsp. infantis	UCD298	2503754025	Human infant feces	ATCC 25962; JCM 1210; DSM 20223
Bifidobacterium longum subsp. *infantis*	UCD299	2503754026	Human infant feces	ATCC 17930; JCM 1260; DSM 20218
Bifidobacterium longum subsp. *infantis*	UCD300	2503754027	Human infant feces	ATCC 15702; JCM 1272; DSM 20090
Bifidobacterium catenulatum subsp. *kashiwanohense*	JCM15439	GCA_001042615.1	Human infant feces	DSM 21854
Bifidobacterium longum subsp. *longum*	JCM1217	GCA_000196555.1	Human feces	
Bifidobacterium longum subsp. *longum*	JCM7052	GCA_015100215.1	Human feces	
Bifidobacterium longum subsp. *longum*	KCTC3128	GCA_017132775.1	Human feces	
Bifidobacterium pseudocatenulatum	DSM20438	GCA_001025215.1	Human infant feces	ATCC 27919; JCM 1200; LMG 10505
Bifidobacterium longum subsp. *suis*	JCM19995	GCA_017132755.1	Piglet feces	DSM 28597
Bifidobacterium longum subsp. *suis*	DSM20211	GCA_000771285.1	Pig feces	
Gardnerella vaginalis	ATCC14018	GCA_001042655.1	Human vaginal secretions	NCTC 10287; JCM 11026

Supplementary Table S1: *Bifidobacterium* strains andassession numbers used to construct the phylogeny.
