# Supplementary material for: Transmission and Persistence of Infant Gut-Associated Bifidobacteria
Source: Microorganisms. 2024 Apr 27;12(5):879. doi: 10.3390/microorganisms12050879 (PMC11124121; doi:10.3390/microorganisms12050879)
Supplement: Supplementary file 1 [file microorganisms-12-00879-s001.zip › Supplementary-text.pdf]

Supplementary Materials:

Materials and methods for phylogenetic inference

Genomic assemblies were retrieved from the NCBI (https://www.ncbi.nlm.nih.gov/) and IMG (https://img.jgi.doe.gov/cgi-bin/m/main.cgi) databases using the GenBank or IMG Genome ID accession numbers listed in Supplementary Table S1. Phylogenetic analysis was performed using the up-to-date bacterial core gene (UBCG2) pipeline, which infers a maximum-likelihood phylogeny using the concatenated sequences of 81 single-copy core genes [103]. UBCG2 utilizes the external programs Prodigal v2.6.3, hmmsearch v3.1, and MAFFT v7.313 in the pipeline [104–106]. Arguments were used to invoke FastTree v2.1.10 for phylogenetic reconstruction and the GTRcat model [107]. *Gardnerella vaginalis* ATCC14018 was used as the outgroup. Phylogenetic trees were visualized and formatted for clarity using the ape, ggtree, glue, ggplot2, and ggtext packages in R [108–112].
